# The colostrum chronicles: identifying porcine milk oligosaccharides in colostrum and investigating their role in litter performance

**DOI:** 10.1093/jas/skag098

**Published:** 2026-03-25

**Authors:** Georgina O Smith, Márton Lengyel, Dora Molnar-Gabor, Henry M R Greathead, Katie McDermott

**Affiliations:** School of Biology, University of Leeds, Leeds, LS2 9JT, United Kingdom; dsm-firmenich, Hørsholm, 2970, Denmark; dsm-firmenich, Hørsholm, 2970, Denmark; School of Biology, University of Leeds, Leeds, LS2 9JT, United Kingdom; School of Biology, University of Leeds, Leeds, LS2 9JT, United Kingdom

**Keywords:** colostrum, litter performance, piglet, porcine milk oligosaccharide, sow

## Abstract

In commercial pig production, sows have been selectively bred to become hyper-prolific. However, litter size is positively associated with pre-weaning mortality rate, often due to factors, such as intrauterine growth restriction, limited teat availability, and unequal access to colostrum. Colostrum plays a critical role in neonatal survival, providing not only passive immunity but also bioactive compounds such as porcine milk oligosaccharides (PMO), which exhibit prebiotic and immunomodulatory properties. While human milk oligosaccharides have been successfully added to infant formula, the potential application of PMO in piglet milk replacers remains underexplored. This study aimed to characterize PMO in colostrum from 45 hyper-prolific Large White × Landrace sows (parity 2–8) and investigate their associations with litter performance metrics, including pre-weaning mortality and piglet body weight. Colostrum was sampled and pooled from multiple teats per sow at the on-set of farrowing. Using LC-MS/MS, 11 PMO structures were identified and analyzed via mixed regression models. Litter size was a significant but modest predictor of lacto-N-neotetraose (LNnT) concentration (*R*^2^ = 0.198; *P* = 0.0143), with concentration decreasing as litter size increased. Higher concentrations of 2′fucosyllactose (2′FL) appeared to reduce the pre-weaning mortality rate, although the significance of this diminished after false discovery rate adjustment. Despite this, our findings suggest that specific PMO play a role in early litter performance. Despite all sows being managed within the same herd, with similar genetics, there was considerable inter-sow variation in colostrum composition. Further research is needed to assess whether supplementing piglet diets with targeted PMO could enhance outcomes in large litters, where competition may render natural concentrations insufficient to exert measurable effects.

## Teaser

This study identifies porcine milk oligosaccharides (PMO) in the colostrum of Large White x Landrace sows and analyses the effects of PMO on litter performance. Results suggest specific structures are impacted by litter size, and others may influence pre-weaning mortality rate.

## Introduction

Colostrum, the first secretion from the mammary gland, plays a critical role in the health and survival of mammalian neonates, including humans, due to the provision of fundamental nutrients and bioactive compounds. Colostrum is responsible for the passive transfer of immunity from sow to piglet and therefore is essential for piglets to consume within 36 h of birth (Bailey et al. 2005; Le Dividich et al. 2005). Early colostrum consumption by the neonate is critical due to containing many bioactive components, including immunoglobulins and milk oligosaccharides, which trigger and support immune development in the piglet (Tao et al. 2010; Zhang et al. 2018). Due to colostrum being vital for piglet survival, anything that restricts a piglet’s intake can lead to major health disturbances. This challenge is particularly pronounced in modern hyper-prolific sows, where large litter sizes increase competition for colostrum.

Colostrum production is highly variable between sows, and volume produced is not associated with the size or weight of the litter (Devillers et al. 2005; Devillers et al. 2007; Quesnel 2011), resulting in piglets from larger litters having reduced access to colostrum. Through selective breeding for reproductive performance, the modern sow has become hyper-prolific, giving birth to 15–17 live piglets per litter in Europe (Hansen 2018; AHDB 2023; Sanz-Fernández et al. 2024). Although this appears to be beneficial from a profit-per-sow perspective, litter size is positively associated with pre-weaning mortality due to factors such as inter-uterine growth restriction (Santos et al. 2022), compromised placental efficiency (Wu et al. 2006), and deficiency in functional teats on the sow (Rohrer and Nonneman 2017; Obermier et al. 2023). Management strategies to reduce mortality often focus on cross-fostering or supplemental milk replacer, however, the former is labour intensive and time sensitive (Zhang et al. 2021) and the latter usually lacks critical bioactive components as evidenced in ingredients of commercial products (AB Neo, personal communication, March 2023; Buitelaar Agriculture, personal communication, Feb 2026; sav-A-caf, 2026). Whilst the importance of immunoglobulins in porcine colostrum has been well studied (summarized by Inoue and Tsukahara [2021]), porcine milk oligosaccharides (PMO) have received less attention.

Milk oligosaccharides can be categorized by various structural components, all with a lactose-reducing end, and usually at least based on sialylation (acidic) or lack thereof (neutral). The basic structure of milk oligosaccharides uses five key constituents: D-glucose (Glc), D-galactose (Gal), N-acetylglucosamine (GlcNAc), L-fucose (Fuc), and N-acetylneuraminic acid (NeuAc) or N-glycolylneuraminic acid (NeuGc); both types of sialic acid, which are used to describe structure using an ordered Hex-HexNAc-Fuc-NeuAc-NeuGc system (where Hex = Hexose [Glc or Gal]), with linkage type and carbon numbers specified where possible. The relevance of these complex and structurally diverse glycans to neonatal nutrition stems from their resistance to digestion in the foregut, allowing them to reach the hindgut intact, where they act as a prebiotic (Gibson and Roberfroid 1995). The fermentation of these structures by resident microbes alters the composition and activity of the hindgut microbiota, triggering downstream effects that elicit benefits, including regulation of intestinal epithelial cells (Perdijk et al. 2019), blocking adhesion of pathogens (Piotrowski et al. 2022), and promoting proliferation of beneficial gut bacteria (Ward et al. 2006).

Anti-pathogenic properties of milk oligosaccharides have been recognized since the 1980s, with human studies on the carcinogenic *Helicobacter pylori* (then recognized as *Campylobacter pylori*) (Evans et al. 1988; Simon et al. 1997). Reduction in pathogens can be enhanced by various characteristics of milk oligosaccharides; the binding of the oligosaccharide to the pathogens directly, thus rendering the binding site unavailable to the epithelium (Newburg et al. 2005); through the upregulation of mucus to increase mucin binding (Baurhoo et al. 2007; Baurhoo et al. 2009); both followed by subsequent elimination via peristalsis; or by increasing proliferation of beneficial bacteria and inducing competitive exclusion. Several pathogens have been tested to identify strengths and limitations in the adhesion characteristics of milk oligosaccharides, including *Escherichia coli* (Facinelli 2019; Wang 2020; Faria et al. 2022) and *Clostridioides difficile* (Piotrowski et al. 2022). Coppa et al. (2006) identified acidic fractions were the most suited to reducing adhesion of a range of pathogens, and while neutral fractions were moderately effective, they did not reduce all strains tested (Coppa et al. 2006).

Beneficial effects of human milk oligosaccharides (HMO) have been well-researched over the last couple of decades (Bode 2012; Triantis et al. 2018; Dinleyici et al. 2023). Since the start of the 20th century, it was recognized that breast milk was important for infant survival, with bottle-fed infants having a seven-fold higher mortality rate (Nützenadel 2010), but it was not until 2016 that HMO were first added to infant formula by a U.S. company (Abbott). Few brands have since followed in adding HMO, creating “gold standard” infant nutrition (Cheng and Yeung 2021; Zhu et al. 2022), and many more opt for supplementing fructo- and/or galacto- oligosaccharides (FOS, GOS, respectively) as a more cost-effective prebiotic alternative. In comparison, the livestock milk formula market is behind; despite a growing demand for supplementary milk to support the large litters of modern hyper-prolific sows (Kobek-Kjeldager et al. 2020; Kobek-Kjeldager et al. 2021; Peltoniemi et al. 2021).

Human milk contains higher concentrations of milk oligosaccharides than most other mammals (Bode 2012; Albrecht et al. 2014), somewhat justifying the rapid progression of HMO inclusion in infant formula; however, there is evidence that supplementation of HMO can improve neonatal and post-wean health of piglets (Wang et al. 2007; Berding et al. 2016; Yin et al. 2019; Luo et al. 2022). Bovine milk-based supplemental milk replacers often also offer oligosaccharide content, as some structures are retained through conjugation to proteins and phospholipids (Barile et al. 2009; Badman et al. 2019). The benefits of bovine colostrum supplementation in place of standard milk replacer of piglets have been reported previously (Huguet et al. 2012; Støy et al. 2014; Sugiharto et al. 2015). Furthermore, bovine colostrum is more readily available than HMO due to being a by-product of the dairy industry (Lallès et al. 2009; Poulsen et al. 2017). Although some oligosaccharide structures are shared between bovine and porcine samples, like HMO, these do not always occur in comparative concentrations (Albrecht et al. 2014). It would therefore be more effective to develop targeted supplements specifically for piglets that boast PMO compositions reflective of what is seen naturally in the sow, as these are likely to offer greater benefits to piglets than human- or bovine-derived components. However, research identifying and quantifying PMO remains comparatively sparse. So far over 90 unique PMO have been reported, compared to over 200 HMO (Wei et al. 2018), and sialylated compounds appear to be the dominant fraction in sow samples (Tao et al. 2010; Cheng et al. 2016; Difilippo et al. 2016; Wei et al. 2018) compared to the majority fucosylated compounds we see in humans. There are reports of significant effects of breed and lactation period on PMO concentrations (Tao et al. 2010; Cheng et al. 2016; Mudd et al. 2016; Trevisi et al. 2020), but understanding of the impact on litter performance is limited. This study aimed to identify and quantify PMO in colostrum of highly productive sows on the same farm, under identical commercial conditions, and explore if PMO profile can impact litter performance parameters. We hypothesized that sialylated structures would dominate across samples and expected to see highly abundant structures, such as 3′-siallylactose (3′SL), 6′-siallylactose (6′SL), and lacto-N-neotetraose (LNnT) as reported by previous studies (Tao et al. 2010; Mudd et al. 2016; Salcedo et al. 2016; Wei et al. 2018; Trevisi et al. 2020). If naturally abundant PMO are associated with improved litter performance, this would strengthen the case for incorporating them as prebiotic additives in piglet milk replacer or creep feed to support neonatal growth, survival, and the weaning transition on farms.

## Materials and methods

All animal practices abided by the DEFRA Code of Practice for the Welfare of Pigs (2020) (DEFRA 2020) and the Welfare of Farmed Animals (England) Regulations (2007). No housing or husbandry practice for this study differed from usual management of farrowing sows at the National Pig Centre (NPC; University of Leeds, Leeds, UK) according to the Council Directive 2008/120/EC. All procedures were reviewed and approved by the NPC-named veterinary surgeon, and ethical approval was received from the animal welfare and ethical review board prior to study commencement. No procedures came under the Animals (Scientific Procedures) Act 1986.

### Sow and piglet management

Sows were multiparous Large White × Landrace housed at the NPC on an indoor production system. Sows at the NPC are kept on a 3-wk batch farrowing system, rotating at 50–60 sows per batch. Dry and gestating sows were kept on straw in a dynamic herd until being moved into individual farrowing pens with temporary crating (JLF15, Jyden, 5.76 m^2^) approximately one week prior to their due date. Sows were secured into crates around 24 h prior to parturition (varying on an individual basis, at the discretion of the stock manager) and released at 4 d post-partum (Radtke et al. 2026). Sows were all fed the same commercial gestating and lactating sow diet following standard commercial practice at the NPC, according to stage of gestation and body condition. Shredded paper (topped up daily) and a wooden block on a chain were offered as enrichment within the farrowing house.

Approximately 24 h post-parturition, piglets were individually ear-tagged using RFID tags, and body weight and sex were recorded. An intramuscular injection of a combined 45 mg toltrazuril and 200 mg iron was administered within 48 h of birth for the prevention of iron deficiency and swine coccidiosis. Individual body weights were recorded again at average day 7 and at average day 28. These body weights were corrected to actual day 7 and actual day 28 weights using day of age at weighing and average daily gain data. Litter size at birth was recorded. All fostering history was recorded along with piglet mortality. Piglets had *ad-libitum* creep feed provided from day seven in addition to sow milk and access to fresh water.

### Colostrum sampling

Samples of colostrum were opportunistically obtained from 45 sows (parity 2–8) by manual manipulation of the teat during parturition, when sows were already crated. Samples were taken as close to onset of parturition as possible. A maximum volume of 10 mL colostrum was taken per sow and pooled from a variety of functioning teats to reduce teat-quality-bias (Ogawa et al. 2014). Colostrum was stored on ice immediately after collection and frozen at −80 °C within approximately 6 h after collection until use.

### Chemicals and reagents

Acetonitrile (HPLC-MS grade) used as an eluent was purchased from TH. Geyer GmbH & Co. KG (Renningen, Germany). High-purity deionized water for eluent preparation and sample treatment (Milli-Q, 18.2 MΩ cm) was obtained from a Nanopure Diamond water purification system (APS Water Services Corporation, Lake Balboa, California, USA). Ammonium formate (HPLC-grade) and HPLC-MS grade 50% formic acid solution in water, used for eluent preparation, were purchased from Honeywell Fluka International Inc. (Charlotte, North Carolina, USA). Anhydrous dimethyl sulfoxide, glacial acetic acid, benzocaine, and picoline borane, for the labeling reaction, were purchased from Merck KGaA (Darmstadt, Germany).

Oligosaccharide standards 2′-fucosyllactose (2′FL), 3′SL, 6′SL, LNnT, and para-lacto-N-neohexaose (pLNnH) (all analytical grade) were used for quantitation, produced by DSM-Firmenich. Their purity was assessed using quantitative NMR with certified reference materials. Panose was used as an internal standard and purchased from Megazyme Ltd. (Wicklow, Ireland). Additional oligosaccharide standards 3′-galactosyllactose (3′GL), lacto-N-neohexaose (LNnH), and sialyllacto-N-neotetraose c (LST-c) were used for peak identification, produced by DSM-Firmenich.

### Oligosaccharide identification and quantification

Colostrum was defrosted in a tube rack at ambient temperature. In a 1.5 mL tube (Eppendorf, Hamburg, Germany) 200 µL colostrum and 200 µL acetonitrile were added. This was vortexed and centrifuged at 17,000 × g in a Thermo Scientific Pico 17 microcentrifuge (Thermo Scientific, Waltham, Massachusetts, USA) for 3 min. The supernatant was decanted into a separate tube and centrifuged again under the same conditions. Samples were then labelled by benzocaine and analyzed by HPLC-UV as described previously (Molnár-Gábor et al. 2024). Briefly, 50 µL of calibration standard, or the colostrum supernatant (as obtained previously), and 50 µL internal standard solution (1 g/L panose) were pipetted into a 1.5 mL microcentrifuge tube, followed by 250 µL of derivatization reagent (prepared from 2 g benzocaine and 5 g picoline borane in 20 mL 70:30 DMSO-acetic acid). The tube was then vortexed and kept in a heating block at 60 °C for 3 h with shaking at 600 rpm (Eppendorf Thermomixer C, Eppendorf AG, Hamberg, Germany). Once finished, tubes were removed and cooled to ambient temperature, 500 µL 9:1 acetonitrile-water was added before vortexing and centrifuging at 17,000 × g for 3 min. The supernatant was analyzed using HPLC (UltiMate 3000 with Accucore Amide HILIC column (150 × 2.1 mm, 2.6 µm), Thermo Fisher Scientific, Waltham, USA) using a gradient of acetonitrile (A) and 10 mM ammonium formate (B) pH adjusted to 3.0 with formic acid. The gradient profile started at 94% A and 6% B, which was held for 0.1 min, and then changed to 88% A and 12% B over 0.1 min. Then it was changed to 80% A and 20% B over 5.8 min, and afterwards, to 60% A and 40% B over 2 min. This was maintained for 3 min, then changed back to the initial eluent composition over 0.1 min. The system was re-equilibrated with this composition for 2.9 min. Detection was by a diode array detector at a wavelength of 300 nm.

Oligosaccharides present in the samples were identified by using reference materials as well as HPLC-MS measurements using conditions identical to the method described above. Underivatized molecular masses were calculated. Where reference materials were not available, exact structures could not be confirmed, therefore, constituent monosaccharides were estimated from the mass (referred to in results as “unconfirmed” structures). A 5-point calibration was prepared from 2′FL, 3′SL, 6′SL, LNnT, and pLNnH for the quantification, and unknown compounds were quantified with the closest calibration peaks. Detector response was taken to be equimolar, as would be expected based on the principle of detection (Bigge et al. 1995; Austin et al. 2019). Total concentration was calculated by adding concentrations of identified PMO together for each sow.

### Statistical analysis

Statistical analysis was conducted using R version 4.5.1 (Posit Software 2025; R Core Team 2025). Data consisted of concentrations of PMO identified (g/L) and total concentration per sow, and performance variables as follows: pre-weaning mortality (%); birth litter size; parity; piglet bodyweight at day 1 (BW1); piglet bodyweight at day 7 (BW7); and piglet bodyweight at day 28 (BW28). Performance variables were analyzed at either sow-level (pre-weaning mortality [%]; litter size; parity) or piglet-level (bodyweight at days 1, 7, and 28 [kg]), with birth sow controlled for when conducting analyses at piglet-level. There was only one sow of parity eight, therefore, this was grouped into parity seven to limit skewing.

Throughout this study, the term “colostrum composition” is used to denote the overall oligosaccharide profile of a colostrum sample, defined by the joint distribution of all quantified oligosaccharides measured in that sample, rather than the concentration of any single oligosaccharide. To investigate colostrum composition effects, a multivariate analysis was used as follows. Concentrations were transformed into relative abundance values and visualized using non-metric multidimensional scaling (NMDS) based on Bray-Curtis distance. Performance variables listed above were fitted to the ordination using the envfit function in the “vegan” package v2.7-1 (Oksanen et al. 2025), which tests for associations between metadata variables and the ordination space via permutation. To formally test the influence of performance variables on overall colostrum composition, a permutational multivariate analysis of variance (PERMANOVA) was conducted with the *adonis2* function in “vegan.” This enabled better control over nested data, which was necessary when looking at the piglet-level performance variables.

For univariate analysis, concentrations of PMO were transformed using the “bestNormalize” package v1.9.1 (Peterson and Cavanaugh 2020; Peterson 2021). Body weight measurements were normally distributed and were used as response variables in mixed-effects models, with the PMO as predictors. In these models, litter size and BW1 were included as covariates, and birth sow was incorporated as a random effect. Models were performed using packages “lme4” (Bates et al. 2015) and “lmerTest” (Kuznetsova et al. 2017).

Pre-weaning mortality exhibited characteristics of a Tweedie distribution and therefore was modeled using a generalized mixed model with a Tweedie family and a log link function with the package “glmmTMB” v1.1.11 (Brooks et al. 2017; McGillycuddy et al. 2025). In this model, PMO were used as predictors, litter size as a covariate, and parity was treated as a random effect. Pre-weaning mortality was analyzed at the sow-level, thus, birth sow as a random effect was unnecessary. Traditional *R*^2^ values for the purpose of understanding how much variation was explained by each PMO are not defined for this model, therefore, the conditional pseudo-*R*^2^ value is used following Nakagawa and Schielzeth (2013) using the package “performance” v0.16.0 (Lüdecke et al. 2021), which gives an appropriate substitution for generalized mixed models reflecting variance explained by both fixed and random factors.

To explore associations between PMO and sow factors, litter size and parity were used as predictors in linear models with the PMO as response variables. Multiple testing correction was performed using a false discovery rate (FDR) approach. Specifically, the Benjamini-Hochberg step-up procedure was used to control the expected proportion of false positives among significant results. Significance was reported at *P* < 0.05 and trending at *P* < 0.1. Regression coefficients are reported as “β.”

## Results

### Oligosaccharide identification and concentration

A total of 11 PMO were identified; five sialylated, five neutral, and one fucosylated compound. Of the 11 found, seven were confirmed by retention times and mass spectrometry of reference compounds. Confirmed structures were 2′-fucosyllactose (2′FL); 3′galactosyllatose (3′GL); 3′-siallylactose (3′SL); 6′-siallylactose (6′SL); lacto-N-neohexaose (LNnH); lacto-N-neotetraose (LNnT); sialyllacto-N-neotetraose c (LSTc). The remaining four were identified based on mass alone, and these unconfirmed structures are reported here as galactosyllactose 1 and 2 (GL1 and GL2), sialyl-lacto-N-triose (S-LN-Tri), and sialyl-lacto-N-neohexaose (SLNnH). See Table 1 for spectroscopy results.

**Table 1:** Porcine milk oligosaccharide structures identified.

Compound name	Compound abbreviation	Retention time, min	Monoisotopic mass	Molecular weight	Structure (where known)^1^
**2′-fucosyllactose**	2′FL	5.63	488.17	488.44	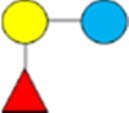
**3**′**-galactosyllatose**	3′GL	7.42	504.17	504.44	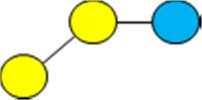
**3**′**-siallylactose**	3′SL	7.61	633.21	633.55	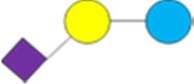
**6**′**-siallylactose**	6′SL	8.81	633.21	633.55	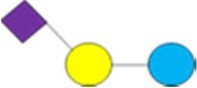
**Galactosyllactose 1**	GL1	7.14	504.17	504.44	
**Galactosyllactose 2**	GL2	7.88	504.17	504.44	
**Sialyl-lacto-N-triose**	S-LN-Tri	8.99	836.29	836.75	
**Lacto-N-neotetraose**	LNnT	9.35	707.25	707.63	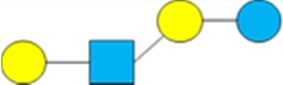
**Sialyllacto-N-neotetraose c**	LST-c	10.40	998.34	998.89	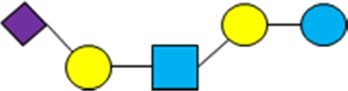
**Lacto-N-neohexaose**	LNnH	10.76	1072.38	1072.97	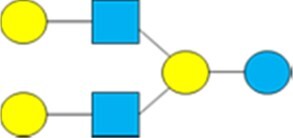
**Sialyl-lacto-N-neohexaose**	SLNnH	11.10	1363.47	1364.22	

1 Diagrams as per Varki et al. (2015) as follows: yellow/light circle is Gal; blue/dark circle is Glc; red triangle is Fuc; purple diamond is Neu5Ac; blue square is GlcNAc.

**Figure 1:**
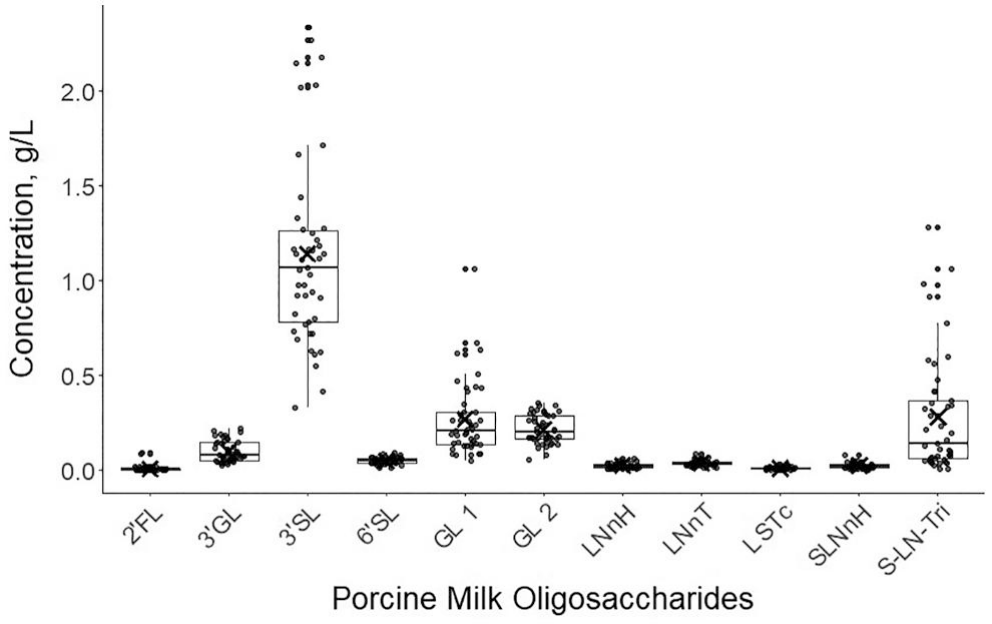
Variation in concentration (g/L) of each identified porcine milk oligosaccharide. Boxes display interquartile range, “whiskers” show range, with line at the median and cross for mean. Abbreviations from left to right: 2′-fucosyllactose (2′FL); 3′galactosyllatose (3′GL); 3′-siallylactose (3′SL); 6′-siallylactose (6′SL); galactosyllactose 1 (GL 1); galactosyllactose 2 (GL 2); lacto-N-neohexaose (LNnH); lacto-N-neotetraose (LNnT); sialyllacto-N-neotetraose c (LSTc); sialyl-lacto-N-neohexaose (SLNnH); sialyl-lacto-N-triose (S-LN-Tri).

Total oligosaccharide concentrations ranged from 0.827 g/L to 4.32 g/L with a mean value of 2.17 ± 0.764 g/L. The most abundant compound across all sows was 3′SL with mean concentration of 1.14 ± 0.498 g/L. The next three most abundant were all unconfirmed compounds; S-LN-Tri (0.282 ± 0.310 g/L), GL1 (0.268 ± 0.197 g/L), and GL2 (0.217 ± 0.0770 g/L). Six of the 11 identified compounds were present in particularly low concentrations (< 0.1 g/L); these were 6′SL, 2′FL, LNnT, LNnH, LSTc, and SLNnH. The concentrations of the identified PMO are presented in Figure 1. Exact concentrations of each PMO for each sow can be seen in Supplementary Material (Table S1).

**Figure 2:**
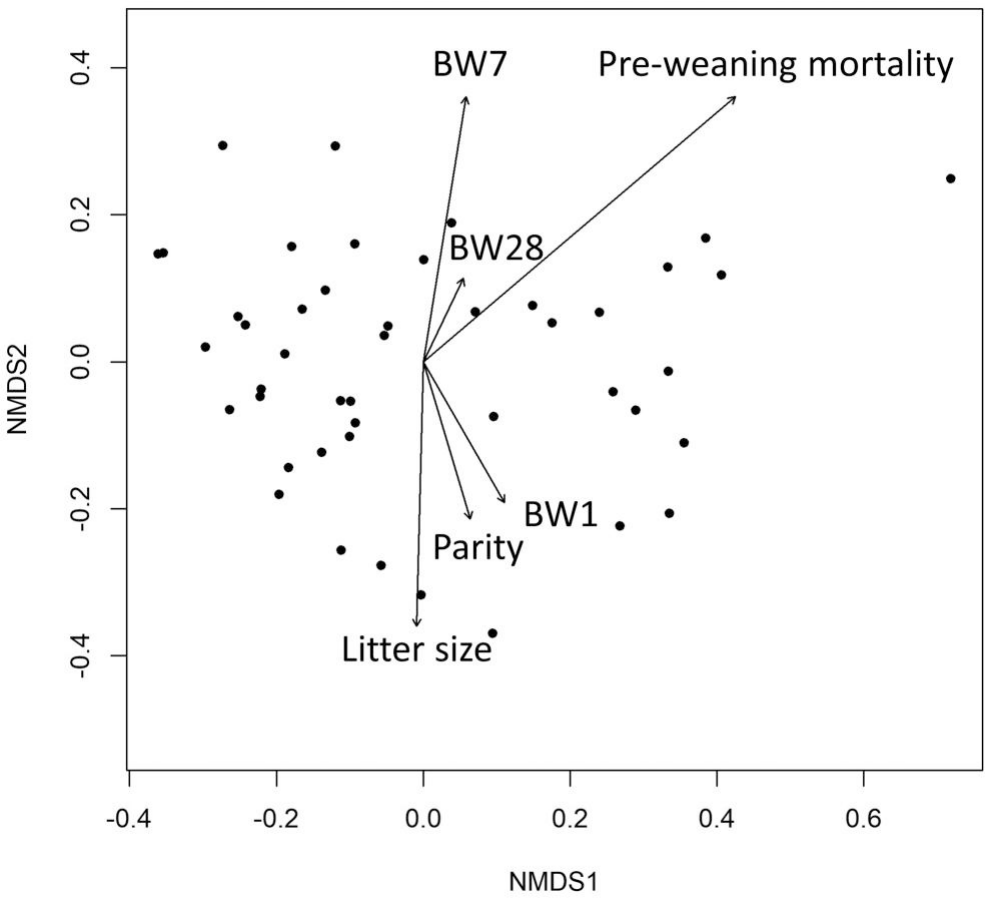
Non-metric multidimensional scaling (NMDS) plot of oligosaccharide profiles based on Bray-Curtis dissimilarities. Each point represents one sow’s colostrum composition. Arrows indicate the direction and strength of associations between the variable and the NMDS axis. No variables were significant (*P* > 0.05); pre-weaning mortality showed a trend (*P* = 0.0560). Variable abbreviations: piglet bodyweight at day one (BW1); piglet bodyweight at day 7 (BW7); and piglet bodyweight at day 28 (BW28).

At the multivariate level, colostrum composition was generally not associated with performance variables (*P* > 0.1), although pre-weaning mortality showed a marginal association (F_1,43_ = 2.87; *P* = 0.0560); the relationships between colostrum composition and performance variables are illustrated in Figure 2.

Univariate analysis for individual oligosaccharide concentrations found no PMO to be significant predictors of BW1, BW7, or BW28 (*P* > 0.1), using mixed-effect linear modeling (all values available in Table S2).

No PMO were significant predictors of pre-weaning mortality rate, although 2′FL concentration was nominally significant prior to FDR correction (*P* = 0.0466), with an increase in 2′FL concentration associated with a decrease in pre-weaning mortality rate (*R*^2^ = 0.332; β = −0.510).

Parity did not significantly predict any PMO concentration. Litter size was a significant predictor of LNnT concentration (*F*_1,43_ = 11.85; *t* = −3.44; *P* = 0.0143), producing a moderate negative regression (*R*^2^ = −0.198). All statistical results for sow-level parameters can be seen in Table S3.

As would be expected, further testing on sow-level parameters showed significant effect of parity (*z* = 2.62; *P* = 0.00869) and litter size (*z* = 2.95; *P* = 0.00323) on prediction of pre-weaning mortality. Although both parity and litter size had positive main effects (β = 1.29 and 0.343, respectively), their interaction term was negative (β = −0.0600; *P* = 0.0208), indicating that the increase in mortality rate associated with larger litter sizes diminishes at higher parities (see Table 2).

**Table 2:** Coefficients of Tweedie model predicting pre-weaning mortality.

Effect	Estimate	Standard error	*Z-*value	*P*-value
**Parity**	1.29	0.492	2.62	0.00869
**Litter size**	0.343	0.116	2.95	0.00323
**Parity: litter size**	−0.0600	0.0259	−2.31	0.0208

## Discussion

Colostrum plays a vital role in neonatal health. The bioactive components of which serve as the first line of immune defense in the first few days after birth. Notably, colostrum yield is not influenced by litter size (Devillers et al. 2005; Devillers et al. 2007; Quesnel 2011), which may result in inadequate intake for individual piglets in larger litters. Given the increased mortality rates observed in large litters (Canario et al. 2006a, 2006b; Rosendo et al. 2007), nutritional supplementation with milk replacers is often considered. However, current piglet milk replacers are suboptimal because they typically lack bioactive components such as prebiotics, which are routinely incorporated into human infant formulas and are important for neonatal gut health. To better inform the development of more optimal milk replacer, the present study aimed to quantify PMO in sow colostrum and investigate their associations with litter performance metrics.

Three of the four most abundant PMO identified were unconfirmed structures (GL1, GL2, and S-LN-Tri), reference to which has been identified in similar abundance in previous porcine literature (Mudd et al. 2016; Wei et al. 2018). The two isomers deemed “galactosyllactose” (GL1 and GL2), we believe, have been identified in porcine samples across other studies (Tao et al. 2010; Salcedo et al. 2016; Wei et al. 2018) based on reported masses and abundances. These trisaccharides are never discussed in depth due to lack of published knowledge on the structures. This may be due to humans lacking milk oligosaccharides with an α1-3 linked galactose (which we assume our GL1 and GL2 have, based on the other porcine studies cited above), and it could be an allergen for human infants (Nguyen et al. 2005; Wilson et al. 2017). Diversity and inconsistency in analytical techniques and nomenclature contribute to this lack of functional knowledge of lesser-known PMO, despite the obvious abundance and frequency in which they are identified. Consequently, comparison between studies is challenging, as many rely on describing general numerical glycan structure (Hex-HexNAc-Fuc-NeuAc-NeuGc) with limited acknowledgement of isomers. Development of techniques and methodologies, such as UPLC-MS/MS or multifaceted approaches, such as described in Hsu et al. (2018) will provide further identification that might enable better discussion regarding their importance in sow colostrum and milk. A recent advance has been made in creating a database for comparison of milk oligosaccharides between mammal species (Durham et al. 2023), but more than anything, this highlights the diversity and scope of unique structures that we need to understand the functionality of.

Prior to FDR adjustment, 2′FL was a statistically significant predictor of pre-weaning mortality. After correction this was not statistically significant, likely due to lack of power and low average concentration (<0.1 g/L), however, this was a pilot study, and it can be suggested that a larger sample size would yield significant results here, particularly with the biological relevance of a negative regression coefficient (−0.510), which would result in a decrease in mortality rate by roughly 40% (based on the Tweedie log link model) per unit (in this study, g/L) increase of 2′FL. The low abundance is corroborated in other porcine studies (Mudd et al. 2016; Wei et al. 2018), and the negative association with pre-weaning mortality may be related to the reported benefits of 2′FL, such as increased proliferation of beneficial gut bacteria (Yu et al. 2013), improved cognitive ability (Oliveros et al. 2016), and reduced inflammation (He et al. 2016). These notable benefits have led to the commercial synthesis and inclusion of 2′FL as a prebiotic in human infant formula with much success (Reverri et al. 2018; Vandenplas et al. 2018).

In humans, the abundance of 2′FL is linked to secretor and Lewis genes for blood typing (Bode 2012). Briefly, those expressing secretor genes at the mammary gland can produce fucosylated milk oligosaccharides, and although no blood testing in these sows is available yet to confirm this, it is a likely explanation for the low concentrations seen. Based on how vast and positive the literature is surrounding 2′FL, and the present preliminary results suggesting a possible effect on pre-weaning mortality, 2′FL may be an obvious compound to target for inclusion in milk replacer formula for piglets to support gut health through its prebiotic activity, particularly when sows generally appear to produce such low concentrations naturally (Mudd et al. 2016; Wei et al. 2018).

Modeling parity as a predictor for the PMO in colostrum did not produce significant results. Parity may not have been a strong enough predictor because it was not a balanced factor in this study due to the opportunistic sampling method; however, other studies concur that parity is not a significant effect regarding PMO (Wei et al. 2018; Trevisi et al. 2020). Wei et al. (2018) do report differences in transitional and mature milk, but much less so in the colostrum of gilts compared to sows. Trevisi et al. (2020) used a mixed-breed population and saw no influence of parity, despite the inclusion of gilt samples. Gilts were excluded from the present study due to concerns they produce less colostrum than multiparous sows (Beyer et al. 2007). Gilts have less developed mammary tissue and mammary DNA (Nielsen et al. 2001), and there is evidence of differences in colostrum composition between primiparous and multiparous sows, such as fat content (Luise et al. 2018; Amatucci et al. 2022). Supplementation of multiparous colostrum to gilt progeny has been shown to reduce the high variability in bodyweights seen in gilt litters and reduce the need for antibiotics (Miguel et al. 2021). However, the majority of literature confirms the main difference to be regarding yield rather than composition (Hansen et al. 2006; Craig et al. 2019; Nuntapaitoon et al. 2020). Yield differences may still explain the benefits observed by Miguel et al. (2021), as the effect appeared to stem from the provision of additional colostrum itself, rather than it being specifically derived from multiparous sows. Lack of parity effect seen in the present study is therefore expected.

Litter size was found to be a significant predictor of LNnT concentration, with the more prolific sows producing less LNnT in their colostrum. Though this suggests some degree of maternal control, the negative association does not support a simple adaptive model. Further possibilities include outpaced physiological optimization and energy cost trade-offs. The former implies that selection for larger litters has accelerated beyond the mammary gland’s adaptive capacity, resulting in suboptimal colostrum composition relative to litter sizes. The latter is logical given the increased energy costs associated with gestating a larger than average litter, as this may lead to prioritization of lactose and macronutrient synthesis over PMO, such as LNnT. While speculative, these possibilities suggest that litter size-associated variation in LNnT may arise from systemic maternal regulation.

The reduction in LNnT concentration seen in larger litters could be a substantial finding due to evidence suggesting how beneficial LNnT is to the immature gut and concerns regarding mortality rates in large litters, also evidenced in this study where every additional piglet resulted in a 0.343% increase in pre-weaning mortality, regardless of parity. The benefits of supplementing LNnT to piglets have been shown through reduction in porcine rotavirus replication in situ (Hester et al. 2013) as well as reduction in duration of diarrhea in rotavirus-infected piglets (Li et al. 2014). Recently, the structural isomer of LNnT, lacto-N-tetraose (LNT), has been added to UK human infant formula (SMA ADVANCED Growing Up Milk|SMA HCP) following the approval of it in Europe as a novel foodstuff (EFSA et al. 2019). The inclusion of LNT rather than LNnT may stem from reports of LNT being a significant human milk component going back to the 1950s (Kuhn 1958). Human milk contains both isomers (Soyyılmaz et al. 2021), while we hypothesize that sow colostrum contains only LNnT as previous porcine studies either confirm the absence of LNT or cannot distinguish between isomers due to methodology limitations (Mudd et al. 2016; Salcedo et al. 2016; Wei et al. 2018).

The difference in structure between the isomers is the linkage type on the terminal galactose (Gal) being β1-3 or β1-4 for LNT and LNnT, respectively. Importantly, both contain the N-acetylglucosamine sugar linked to this terminal Gal, which is valued due to having the “Bifidus Factor”; meaning this encourages growth of *Bifidobacterium spp.* (Cordeiro et al. 2023; Li et al. 2023), many of which are beneficial to the human and piglet gut alike (Pang et al. 2022; He et al. 2023). In human breastfed infants, *Bifidobacterium spp*. typically dominate the gut microbiota and are strongly associated with HMO utilization (Sela and Mills 2010; Zhang et al. 2021). Piglets generally have lower baseline *Bifidobacterium spp*. abundance than human infants (Wang and Donovan 2015), and it is important to acknowledge that the ecological relevance of bifidogenic effects will therefore differ between the species. Although the functional consequences of LNnT on *Bifidobacterium spp*. proliferation in piglets may not be parallel to those observed in humans, benefits are reported following probiotic supplementation of *Bifidobacterium spp*. suggest that promoting these species may nonetheless contribute positively to gut health in the piglet (Hansen et al. 2022; Hansen et al. 2025).

Additionally, N-acetylglucosamine has been shown to enhance multiple gut barrier functions in a mouse model, such as increasing expression of tight junction proteins and reducing loss of mucin-secreting areas (Choi et al. 2023). The immunomodulatory and prebiotic effects of LNnT could be harnessed through the addition of LNnT to a supplemental milk formula to reduce pre-weaning mortality rates via improved piglet gut health and immunity. Litter size was not a predictor of any other individual PMO, nor was it significantly associated with colostrum composition, which aligns with other findings suggesting colostrum quantity is not impacted by number of piglets (Devillers et al. 2005; Devillers et al. 2007; Quesnel 2011). It is an interesting phenomenon and affirms the need for supplemental milk in large litters. Therefore, LNnT is a PMO of interest for developing supplemental milk for piglets, which may improve mortality outcomes on farms.

Lack of significance for longer-term performance parameters, such as BW7 and BW28 was expected, as mature milk is the largest provision of nutrients after the first day of lactation, and so lasting performance effects from colostrum would be exceptional. Colostrum was analyzed in this study due to the pilot nature of this work and the practical and ethical simplicity relative to collecting mature milk, which often requires injection of oxytocin (Tao et al. 2010; Mudd et al. 2016). Further research should certainly explore litter performance traits associated with PMO concentrations in mature milk at various points in lactation to fully understand how these structures affect piglet health and performance longitudinally. This work could additionally incorporate the study of teat fidelity, as samples in the present study were pooled to reduce teat influence. Quality differs between teats (Ogawa et al. 2014), and there is already evidence suggesting anterior teat sucklers weigh more at weaning than piglets suckling posterior teats (Dierking et al. 2025; Mata et al. 2025). Furthermore, piglets show loyalty to a preferred teat throughout the suckling period (De Passillé et al. 1988) as well as awareness regarding teat productivity (Devillers et al. 2016), therefore, links between teat selection and piglet performance would be interesting to explore.

The quantification method used in the present study was based on work by Molnár-Gábor et al. (2024) with modifications made to adapt the protocol to the use of colostrum. Total solids, fat, and lactose values can be similar between milk and colostrum, depending on the lactation period (Hurley 2015), but one of the largest dissimilarities between the matrices is the immunoglobulin content, which is significantly higher in colostrum (Hurley and Theil 2013). During sample preparation, proteins are precipitated out of the matrix using acetonitrile, with samples being processed once the supernatant is clear enough, thus minimizing risk of any contamination. It is generally accepted to prepare samples in a similar manner as long as the matrices are broadly similar, for example, colostrum, mature milk, or other liquid feeds (Tao et al. 2009; Fischer-Tlustos et al. 2020; Bunyatratchata et al. 2021). Furthermore, identically prepared cross-species milk samples are often compared without performing methodological changes for each matrix (Albrecht et al. 2014; Urrutia-Baca et al. 2023), despite milk compositional differences having been reported between species (Roy et al. 2020). The derivatization procedure in the present study relies on the reaction between the labeling agent (benzocaine) and the formyl group of each PMO. Labeled PMO are then separated via HPLC using a technique that favors hydrophilic compounds. Interference using this method is low, as compounds would need to meet all the following criteria to pose a risk: remain in the aqueous supernatant during sample preparation; contain a formyl group or coincidentally absorb at the wavelength used; and be sufficiently hydrophilic to be retained on the chromatographic column to an extent comparable to PMO. Therefore, the analytical method should be broadly applicable across various milk-adjacent substrates.

Beyond agricultural relevance, the neonatal pig is widely recognized as a translational model for human infant nutrition and development. Pre-clinical studies using piglets have demonstrated that supplementation with milk oligosaccharides, including 2′FL and LNnT, can modulate gut microbial composition (Li et al. 2014; Wang et al. 2021), enhance intestinal barrier function (Berding et al. 2016; Yin et al. 2019), and impact aspects of neurodevelopment (Wang et al. 2007). These findings contribute to the evidence base supporting the inclusion of these compounds in milk formula. In this context, characterizing the natural variation of PMO and their associations with piglet outcomes not only informs swine production but also strengthens interpretation of the pig as a biomedical model for early life nutritional programming.

In conclusion, PMO concentrations are highly individual to each sow, regardless of parity, despite controlled breeding, feeding, and environment. No PMO significantly impacted bodyweights at any time point. Higher 2′FL concentration may reduce pre-weaning mortality, but a larger-powered study is necessary to confirm this. Concentration of LNnT was negatively associated with litter size, suggesting this may be a good target for supplementation given the beneficial effects reported in literature and the increase of pre-weaning mortality with litter size. Both 2′FL and a structural isomer of LNnT have been included in human infant formula with success, therefore, consideration should be made for the pig industry to adopt similar advances.

## Supplementary Material

skag098_Supplementary_Data

## Data Availability

Data can be made available from the corresponding author upon reasonable request.

## Abbreviations

2′FL: 2′-fucosyllactose
3′GL: 3′galactosyllatose
3′SL: 3′-siallylactose
6′SL: 6′-siallylactose
BW1: piglet bodyweight at day 1
BW28: piglet bodyweight at day 28
BW7: piglet bodyweight at day 7
DEFRA: Department for Environment, Food and Rural Affairs
FDR: false discovery rate
FOS: fructooligosaccharides
Fuc: L-fucose
Gal: D-galactose
Glc: D-glucose
GlcNAc: N-acetylglucosamine
GOS: Galactooligosaccharides
GL1: Galactosyllactose 1
GL2: Galactosyllactose 2
Hex: hexose
HMO: human milk oligosaccharides
LNnH: lacto-N-neohexaose
LNnT: lacto-N-neotetraose
LNT: lacto-N-tetraose
LSTc: sialyllacto-N-neotetraose c
NeuAc: N-acetylneuraminic acid
NeuGc: N-glycolylneuraminic acid
NPC: National Pig Centre
pLNnH: Para-lacto-N-neohexaose
PMO: porcine milk oligosaccharides
SLNnH: sialyl-lacto-N-neohexaose
S-LN-Tri: sialyl-lacto-N-triose
