# Supplementary material for: The colostrum chronicles: identifying porcine milk oligosaccharides in colostrum and investigating their role in litter performance
Source: J Anim Sci. 2026 Mar 25;104:skag098. doi: 10.1093/jas/skag098 (PMC13181254; doi:10.1093/jas/skag098)
Supplement: skag098_Supplementary_Data [file skag098_supplementary_data.zip › Supp. Table 1.docx]

# Supplementary Table 1 Porcine milk oligosaccharide concentrations (g/L) per sow

| **SowID** | **Concentration, g/L** | | | | | | | | | | | | |
| --- | --- | --- | --- | --- | --- | --- | --- | --- | --- | --- | --- | --- | --- |
|  | **2'FL** | **3'GL** | **GSL 1** | **GSL 2** | **3'SL** | **6'SL** | **S-LN-Tri** | **LNnT** | **LSTc** | **LNnH** | **SLNnH** | **Total** |  |
| 220 | 0.014 | 0.075 | 0.472 | 0.172 | 1.332 | 0.066 | 0.062 | 0.042 | 0.013 | 0.013 | 0.018 | 2.279 |  |
| 310 | 0.000 | 0.203 | 0.673 | 0.310 | 2.176 | 0.067 | 0.776 | 0.033 | 0.016 | 0.031 | 0.032 | 4.318 |  |
| 311 | 0.008 | 0.098 | 1.063 | 0.213 | 1.663 | 0.089 | 0.286 | 0.023 | 0.008 | 0.021 | 0.034 | 3.505 |  |
| 551 | 0.003 | 0.026 | 0.239 | 0.056 | 0.413 | 0.017 | 0.047 | 0.014 | 0.000 | 0.007 | 0.005 | 0.827 |  |
| 552 | 0.009 | 0.084 | 0.196 | 0.169 | 1.108 | 0.044 | 0.232 | 0.027 | 0.006 | 0.013 | 0.010 | 1.898 |  |
| 560 | 0.003 | 0.151 | 0.191 | 0.279 | 1.265 | 0.067 | 0.979 | 0.038 | 0.016 | 0.041 | 0.045 | 3.075 |  |
| 574 | 0.003 | 0.109 | 0.350 | 0.258 | 1.184 | 0.063 | 0.082 | 0.040 | 0.004 | 0.041 | 0.038 | 2.172 |  |
| 576 | 0.006 | 0.097 | 0.086 | 0.212 | 0.622 | 0.065 | 1.279 | 0.038 | 0.022 | 0.034 | 0.047 | 2.509 |  |
| 593 | 0.009 | 0.220 | 0.140 | 0.345 | 2.268 | 0.067 | 0.326 | 0.035 | 0.007 | 0.048 | 0.053 | 3.518 |  |
| 636 | 0.003 | 0.069 | 0.208 | 0.141 | 1.165 | 0.036 | 0.111 | 0.037 | 0.008 | 0.021 | 0.018 | 1.815 |  |
| 642 | 0.003 | 0.172 | 0.122 | 0.287 | 1.248 | 0.061 | 1.059 | 0.064 | 0.027 | 0.061 | 0.081 | 3.185 |  |
| 644 | 0.005 | 0.208 | 0.084 | 0.343 | 2.333 | 0.059 | 0.076 | 0.034 | 0.006 | 0.025 | 0.026 | 3.198 |  |
| 719 | 0.010 | 0.073 | 0.432 | 0.223 | 2.029 | 0.061 | 0.132 | 0.025 | 0.012 | 0.024 | 0.023 | 3.045 |  |
| 743 | 0.003 | 0.048 | 0.239 | 0.182 | 0.919 | 0.079 | 0.099 | 0.060 | 0.015 | 0.020 | 0.026 | 1.690 |  |
| 759 | 0.019 | 0.143 | 0.637 | 0.304 | 1.212 | 0.057 | 0.917 | 0.039 | 0.020 | 0.023 | 0.029 | 3.401 |  |
| 761 | 0.093 | 0.065 | 0.052 | 0.320 | 0.976 | 0.069 | 0.009 | 0.012 | 0.010 | 0.036 | 0.022 | 1.663 |  |
| 765 | 0.013 | 0.087 | 0.141 | 0.173 | 1.069 | 0.048 | 0.036 | 0.028 | 0.006 | 0.011 | 0.010 | 1.622 |  |
| 855 | 0.000 | 0.078 | 0.265 | 0.146 | 0.974 | 0.035 | 0.580 | 0.012 | 0.000 | 0.016 | 0.015 | 2.120 |  |
| 858 | 0.022 | 0.119 | 0.215 | 0.255 | 1.163 | 0.044 | 0.196 | 0.072 | 0.018 | 0.028 | 0.031 | 2.162 |  |
| 860 | 0.000 | 0.051 | 0.111 | 0.136 | 0.608 | 0.036 | 0.289 | 0.037 | 0.014 | 0.018 | 0.018 | 1.317 |  |
| 862 | 0.005 | 0.076 | 0.265 | 0.203 | 0.769 | 0.048 | 0.043 | 0.036 | 0.006 | 0.017 | 0.016 | 1.484 |  |
| 873 | 0.000 | 0.147 | 0.158 | 0.312 | 0.924 | 0.060 | 0.077 | 0.037 | 0.015 | 0.028 | 0.022 | 1.781 |  |
| 876 | 0.000 | 0.152 | 0.090 | 0.262 | 2.144 | 0.072 | 0.050 | 0.048 | 0.008 | 0.042 | 0.040 | 2.909 |  |
| 908 | 0.007 | 0.042 | 0.134 | 0.124 | 0.908 | 0.052 | 0.008 | 0.033 | 0.008 | 0.012 | 0.012 | 1.339 |  |
| 916 | 0.000 | 0.186 | 0.192 | 0.293 | 1.271 | 0.062 | 0.106 | 0.041 | 0.011 | 0.029 | 0.027 | 2.218 |  |
| 918 | 0.008 | 0.071 | 0.432 | 0.173 | 0.630 | 0.055 | 0.419 | 0.022 | 0.000 | 0.010 | 0.010 | 1.829 |  |
|  |  |  |  |  |  |  |  |  |  |  |  |  |  |
| **SowID** | **Concentration, g/L** | | | | | | | | | | | |  |
|  | **2'FL** | **3'GL** | **GSL 1** | **GSL 2** | **3'SL** | **6'SL** | **S-LN-Tri** | **LNnT** | **LSTc** | **LNnH** | **SLNnH** | **Total** |  |
| 919 | 0.002 | 0.052 | 0.130 | 0.175 | 1.142 | 0.053 | 0.162 | 0.034 | 0.010 | 0.023 | 0.021 | 1.803 |  |
| 926 | 0.000 | 0.180 | 0.135 | 0.326 | 1.438 | 0.075 | 0.353 | 0.057 | 0.010 | 0.047 | 0.049 | 2.670 |  |
| 958 | 0.000 | 0.062 | 0.443 | 0.135 | 0.689 | 0.046 | 0.368 | 0.017 | 0.000 | 0.010 | 0.016 | 1.787 |  |
| 965 | 0.007 | 0.050 | 0.180 | 0.144 | 0.551 | 0.052 | 0.416 | 0.030 | 0.011 | 0.014 | 0.017 | 1.472 |  |
| 973 | 0.006 | 0.122 | 0.187 | 0.242 | 1.053 | 0.036 | 0.345 | 0.047 | 0.009 | 0.030 | 0.024 | 2.099 |  |
| 997 | 0.003 | 0.110 | 0.146 | 0.176 | 0.783 | 0.028 | 0.336 | 0.030 | 0.021 | 0.018 | 0.013 | 1.664 |  |
| 1017 | 0.010 | 0.164 | 0.164 | 0.301 | 0.718 | 0.042 | 0.600 | 0.045 | 0.019 | 0.040 | 0.034 | 2.136 |  |
| 1029 | 0.000 | 0.050 | 0.614 | 0.170 | 0.731 | 0.076 | 0.141 | 0.042 | 0.011 | 0.019 | 0.030 | 1.884 |  |
| 1070 | 0.003 | 0.035 | 0.133 | 0.081 | 0.333 | 0.015 | 0.217 | 0.018 | 0.000 | 0.010 | 0.008 | 0.852 |  |
| 1071 | 0.018 | 0.049 | 0.218 | 0.167 | 0.719 | 0.036 | 0.068 | 0.052 | 0.009 | 0.012 | 0.011 | 1.358 |  |
| 1073 | 0.011 | 0.041 | 0.506 | 0.134 | 0.941 | 0.040 | 0.065 | 0.045 | 0.012 | 0.015 | 0.019 | 1.828 |  |
| 1076 | 0.020 | 0.144 | 0.082 | 0.240 | 1.713 | 0.041 | 0.562 | 0.045 | 0.018 | 0.031 | 0.034 | 2.931 |  |
| 1092 | 0.088 | 0.091 | 0.090 | 0.306 | 0.800 | 0.038 | 0.055 | 0.025 | 0.006 | 0.053 | 0.020 | 1.572 |  |
| 1096 | 0.007 | 0.183 | 0.309 | 0.289 | 2.015 | 0.091 | 0.044 | 0.058 | 0.034 | 0.028 | 0.045 | 3.100 |  |
| 1101 | 0.002 | 0.189 | 0.309 | 0.353 | 1.033 | 0.064 | 0.476 | 0.089 | 0.027 | 0.050 | 0.046 | 2.639 |  |
| 1164 | 0.020 | 0.039 | 0.419 | 0.121 | 1.156 | 0.029 | 0.090 | 0.045 | 0.010 | 0.012 | 0.016 | 1.956 |  |
| 1184 | 0.010 | 0.064 | 0.258 | 0.189 | 1.142 | 0.055 | 0.026 | 0.063 | 0.019 | 0.012 | 0.018 | 1.856 |  |
| 1191 | 0.013 | 0.057 | 0.266 | 0.169 | 1.118 | 0.035 | 0.036 | 0.043 | 0.008 | 0.014 | 0.017 | 1.775 |  |
| 1192 | 0.009 | 0.047 | 0.295 | 0.172 | 0.824 | 0.046 | 0.039 | 0.035 | 0.016 | 0.013 | 0.014 | 1.510 |  |
