# Supplementary material for: The colostrum chronicles: identifying porcine milk oligosaccharides in colostrum and investigating their role in litter performance
Source: J Anim Sci. 2026 Mar 25;104:skag098. doi: 10.1093/jas/skag098 (PMC13181254; doi:10.1093/jas/skag098)
Supplement: skag098_Supplementary_Data [file skag098_supplementary_data.zip › Supp. Table 2.docx]

Supplementary Table 2 Summary of univariate mixed-effects models testing associations between individual PMOs and piglet level performance variables

| **Performance variable** | **PMO^1^** | **Estimate** | **Test statistic** | **Raw P value** | **FDR^2^ adjusted P value** |
| --- | --- | --- | --- | --- | --- |
| Piglet bodyweight at day one | 2'FL | 0.00771 | 0.262 | 0.795 | 0.795 |
|  | 3'GL | -0.0405 | -1.52 | 0.137 | 0.302 |
|  | 3'SL | -0.0496 | -1.83 | 0.0754 | 0.259 |
|  | 6'SL | -0.0335 | -1.22 | 0.230 | 0.421 |
|  | LNnH | -0.0457 | -1.71 | 0.0943 | 0.259 |
|  | LNnT | -0.0087 | -0.281 | 0.780 | 0.795 |
|  | LSTc | -0.0207 | -0.697 | 0.490 | 0.673 |
|  | GL1 | 0.0146 | 0.511 | 0.612 | 0.748 |
|  | GL2 | -0.046 | -1.74 | 0.0891 | 0.259 |
|  | S-LN-Tri | -0.0281 | -1.03 | 0.310 | 0.488 |
|  | SLNnH | -0.0459 | -1.72 | 0.0932 | 0.259 |
| Piglet bodyweight at day seven | 2'FL | 0.0511 | 1.17 | 0.250 | 0.714 |
|  | 3'GL | 0.0347 | 0.839 | 0.406 | 0.714 |
|  | 3'SL | -0.0177 | -0.413 | 0.681 | 0.833 |
|  | 6'SL | -0.00252 | -0.0600 | 0.953 | 0.953 |
|  | LNnH | 0.0313 | 0.755 | 0.455 | 0.714 |
|  | LNnT | -0.0441 | -0.951 | 0.347 | 0.714 |
|  | LSTc | 0.028 | 0.623 | 0.537 | 0.738 |
|  | GL1 | -0.0839 | -2.03 | 0.0493 | 0.543 |
|  | GL2 | 0.0523 | 1.29 | 0.206 | 0.714 |
|  | S-LN-Tri | -0.0454 | -1.10 | 0.279 | 0.714 |
|  | SLNnH | -0.0128 | -0.306 | 0.761 | 0.838 |

| **Performance variable** | **PMO^1^** | **Estimate** | **Test statistic** | **Raw P value** | **FDR adjusted P value** |
| --- | --- | --- | --- | --- | --- |
| Piglet bodyweight at day 28 | 2'FL | 0.134 | 1.00 | 0.323 | 0.977 |
|  | 3'GL | 0.0261 | 0.204 | 0.839 | 0.977 |
|  | 3'SL | -0.00381 | -0.029 | 0.977 | 0.977 |
|  | 6'SL | 0.00709 | 0.055 | 0.956 | 0.977 |
|  | LNnH | 0.0648 | 0.507 | 0.615 | 0.977 |
|  | LNnT | -0.224 | -1.62 | 0.113 | 0.977 |
|  | LSTc | -0.00525 | -0.038 | 0.970 | 0.977 |
|  | GL1 | -0.159 | -1.23 | 0.228 | 0.977 |
|  | GL2 | 0.0657 | 0.518 | 0.607 | 0.977 |
|  | S-LN-Tri | -0.061 | -0.473 | 0.638 | 0.977 |
|  | SLNnH | 0.0117 | 0.091 | 0.928 | 0.977 |

^1^Porcine milk oligosaccharide: 2’-fucosyllactose (2’FL); 3’galactosyllatose (3’GL); 3’-siallylactose (3’SL); 6’-siallylactose (6’SL); lacto-N-neohexaose (LNnH); lacto-N-neotetraose (LNnT); sialyllacto-N-neotetraose c (LSTc); galactosyllactose 1(GL1); galactosyllactose 2 (GL2); sialyl-lacto-N-triose (S-LN-Tri); sialyl-lacto-N-neohexaose (SLNnH)

^2^False discovery rate using the Benjamini-Hochberg step-up procedure
