# Supplementary material for: The colostrum chronicles: identifying porcine milk oligosaccharides in colostrum and investigating their role in litter performance
Source: J Anim Sci. 2026 Mar 25;104:skag098. doi: 10.1093/jas/skag098 (PMC13181254; doi:10.1093/jas/skag098)
Supplement: skag098_Supplementary_Data [file skag098_supplementary_data.zip › Supp. Table 3.docx]

Supplementary Table 3 Summary of univariate mixed-effects models testing associations between individual PMOs and sow level performance variables

| **Performance variable** | **PMO^1^** | **Estimate** | **Test statistic** | **Adjusted R-squared** | **Raw P value** | **FDR^3^ adjusted P value** |
| --- | --- | --- | --- | --- | --- | --- |
| Pre-weaning mortality^2^ | 2'FL | -0.510 | -1.99 | 0.332 | 0.0466 | 0.435 |
|  | 3'GL | 0.647 | 1.18 | 0.250 | 0.237 | 0.435 |
|  | 3'SL | 0.0666 | 0.102 | 0.238 | 0.919 | 0.919 |
|  | 6'SL | 2.18 | 0.650 | 0.235 | 0.516 | 0.634 |
|  | LNnH | 0.615 | 1.06 | 0.244 | 0.288 | 0.452 |
|  | LNnT | -15.4 | -1.28 | 0.299 | 0.202 | 0.435 |
|  | LSTc | -6.27 | -0.359 | 0.244 | 0.719 | 0.791 |
|  | GL1 | -0.661 | -1.48 | 0.299 | 0.139 | 0.435 |
|  | GL2 | 0.0850 | 0.646 | 0.235 | 0.518 | 0.634 |
|  | S-LN-Tri | 0.208 | 1.70 | 0.270 | 0.0899 | 0.435 |
|  | SLNnH | 0.741 | 1.31 | 0.249 | 0.191 | 0.435 |
| Birth litter size | 2'FL | 0.00281 | 0.127 | -0.0229 | 0.899 | 0.932 |
|  | 3'GL | -0.000927 | -0.086 | -0.0231 | 0.932 | 0.932 |
|  | 3'SL | 0.00473 | 0.573 | -0.0155 | 0.570 | 0.776 |
|  | 6'SL | -0.000874 | -0.506 | -0.0172 | 0.616 | 0.776 |
|  | LNnH | -0.00812 | -0.797 | -0.00835 | 0.430 | 0.776 |
|  | LNnT | -0.00163 | -3.44 | 0.198 | 0.00130 | 0.0143 |
|  | LSTc | -0.000657 | -2.08 | 0.0700 | 0.0438 | 0.241 |
|  | GL1 | 0.0154 | 1.26 | 0.0129 | 0.216 | 0.776 |
|  | GL2 | -0.0362 | -0.836 | -0.00688 | 0.408 | 0.776 |
|  | S-LN-Tri | 0.0216 | 0.478 | -0.0179 | 0.635 | 0.776 |
|  | SLNnH | -0.00915 | -0.853 | -0.00622 | 0.398 | 0.776 |

| **Performance variable** | **PMO** | **Estimate** | **Test statistic** | **Adjusted R-squared** | **Raw P value** | **FDR adjusted P value** |
| --- | --- | --- | --- | --- | --- | --- |
| Parity | 2'FL | -0.037 | -0.861 | -0.0059 | 0.394 | 0.619 |
|  | 3'GL | 0.0141 | 0.671 | -0.0127 | 0.506 | 0.696 |
|  | 3'SL | 0.0187 | 1.17 | 0.00812 | 0.250 | 0.507 |
|  | 6'SL | 0.00533 | 1.61 | 0.035 | 0.115 | 0.507 |
|  | LNnH | -0.00204 | -0.100 | -0.023 | 0.921 | 0.921 |
|  | LNnT | -0.00147 | -1.43 | 0.023 | 0.161 | 0.507 |
|  | LSTc | -0.000717 | -1.12 | 0.00556 | 0.271 | 0.507 |
|  | GL1 | 0.0266 | 1.10 | 0.00485 | 0.277 | 0.507 |
|  | GL2 | -0.0106 | -0.120 | -0.0229 | 0.902 | 0.921 |
|  | S-LN-Tri | 0.105 | 1.20 | 0.00979 | 0.237 | 0.507 |
|  | SLNnH | 0.00539 | 0.254 | -0.0217 | 0.801 | 0.921 |

^1^Porcine milk oligosaccharide: 2’-fucosyllactose (2’FL); 3’galactosyllatose (3’GL); 3’-siallylactose (3’SL); 6’-siallylactose (6’SL); lacto-N-neohexaose (LNnH); lacto-N-neotetraose (LNnT); sialyllacto-N-neotetraose c (LSTc); galactosyllactose 1(GL1); galactosyllactose 2 (GL2); sialyl-lacto-N-triose (S-LN-Tri); sialyl-lacto-N-neohexaose (SLNnH)

^2^Adjusted R-squared for pre-weaning mortality are conditional pseudo-R-squared values following Nakagawa and Schielzeth (2012)

^3^False discovery rate using the Benjamini-Hochberg step-up procedure
